# Decreased glutathione biosynthesis contributes to EGFR T790M-driven erlotinib resistance in non-small cell lung cancer

**DOI:** 10.1038/celldisc.2016.31

**Published:** 2016-09-27

**Authors:** Hongde Li, William Stokes, Emily Chater, Rajat Roy, Elza de Bruin, Yili Hu, Zhigang Liu, Egbert F Smit, Guus JJE Heynen, Julian Downward, Michael J Seckl, Yulan Wang, Huiru Tang, Olivier E Pardo

**Affiliations:** 1 State Key Laboratory of Genetic Engineering, Ministry of Education Key Laboratory of Contemporary Anthropology, Collaborative Innovation Centre for Genetics and Development, Shanghai International Centre for Molecular Phenomics, Metabonomics and Systems Biology Laboratory, Department of Biochemistry, School of Life Sciences, Zhongshan Hospital, Fudan University, Shanghai, China; 2 Key Laboratory of Magnetic Resonance in Biological Systems, National Centre for Magnetic Resonance in Wuhan, State Key Laboratory of Magnetic Resonance and Atomic and Molecular Physics, Wuhan Institute of Physics and Mathematics, Chinese Academy of Sciences, Wuhan, China; 3 Division of Cancer, Department of Surgery and Cancer, Imperial College, Hammersmith Hospital, London, UK; 4 Signal Transduction Laboratory, CRUK London Research Institute, London, UK; 5 Personalised Healthcare & Biomarkers, Innovative Medicines and Early Development Biotech Unit, AstraZeneca, Cambridge, UK; 6 Department of Pulmonary Diseases, VU University Medical Centre, Netherlands Cancer Institute, Amsterdam, Netherlands; 7 Section of Molecular Carcinogenesis, Netherlands Cancer Institute, Amsterdam, Netherlands; 8 Collaborative Innovation Centre for Diagnosis and Treatment of Infectious Diseases, Hangzhou, China

**Keywords:** metabolomics, glutathione, lung cancer, drug resistance, TKI

## Abstract

Epidermal growth factor receptor (EGFR) inhibitors such as erlotinib are novel effective agents in the treatment of EGFR-driven lung cancer, but their clinical impact is often impaired by acquired drug resistance through the secondary T790M EGFR mutation. To overcome this problem, we analysed the metabonomic differences between two independent pairs of erlotinib-sensitive/resistant cells and discovered that glutathione (GSH) levels were significantly reduced in T790M EGFR cells. We also found that increasing GSH levels in erlotinib-resistant cells re-sensitised them, whereas reducing GSH levels in erlotinib-sensitive cells made them resistant. Decreased transcription of the GSH-synthesising enzymes (GCLC and GSS) due to the inhibition of NRF2 was responsible for low GSH levels in resistant cells that was directly linked to the T790M mutation. T790M EGFR clinical samples also showed decreased expression of these key enzymes; increasing intra-tumoural GSH levels with a small-molecule GST inhibitor re-sensitised resistant tumours to erlotinib in mice. Thus, we identified a new resistance pathway controlled by EGFR T790M and a therapeutic strategy to tackle this problem in the clinic.

## Introduction

Lung cancer is the main cancer killer and non-small cell lung cancer (NSCLC) represents ~85% of such cases. About 10% and 30% of NSCLCs in Western and Asian populations, respectively, express an activated mutant epidermal growth factor receptor (EGFRm) and the vast majority (90%) of such patients respond to ATP-competitive EGFR tyrosine kinase inhibitors (TKIs) such as gefitinib or erlotinib [1–3]. Unfortunately, most patients can quickly acquire TKI resistance limiting the benefits of these drugs to patients' survival.

Resistance mechanisms include Met amplification (~5% of cases) [4] and more frequently (50% of cases) an additional T790M gatekeeper mutation within the EGFRm [5–8]. The latter enhances kinase activity by increasing the affinity of EGFR for ATP, competing out TKI binding [9]. This led to the development of compounds irreversibly interacting with EGFRm/T790M, such as afatinib, 324674 and more recently AZD9291 and CO1696 [10–12]. However, thus far clinical trials of afatinib failed to demonstrate improved response in EGFRm/T790M patients [13], and although initial trials with the irreversible inhibitor AZD9291 showed great promises, additional resistance mechanisms to these inhibitors have already surfaced [14].

Changes in cellular metabolism accompany tumourigenesis and classical chemoresistance [15–17]. Hence, changes in metabolite concentrations can specifically reflect the onset of therapy resistance, providing response/outcome biomarkers and novel therapeutic strategies to reverse resistance [18, 19]. Both ^1^H-nuclear magnetic resonance (NMR) and mass spectrometry are efficient tools to investigate these metabolic changes [20–23].

Here we used ^1^H-NMR to compare the metabolic signatures of paired NSCLC cell lines expressing EGFRm without (erlotinib sensitive) or with the additional T790M mutation (erlotinib resistant). We showed that glutathione (GSH) levels were reduced in erlotinib-resistant NSCLC cells in a T790M-dependent manner due to the decreased expression of GSH-synthesising enzymes. Correcting this defect re-sensitised resistant cells to erlotinib *in vitro* and *in vivo*. Moreover, ethacrynic acid (EA), a glutathione-*S*-transferase inhibitor, reversed erlotinib resistance in T790M NSCLC cells *in vitro* and *in vivo* by increasing GSH levels. As EA is a clinically used diuretic, it could be repurposed to reverse T790M-mediated erlotinib resistance in NSCLC patients. Overall, our work demonstrated the power of metabonomic screening to generate novel research hypotheses and discover unexplored strategies to tackle drug resistance in lung cancer treatments.

## Results

### ^1^H-NMR-based metabolic profiling reveals decreased GSH levels in erlotinib-resistant NSCLC cells

Two pairs of cell lines were employed to obtain generic metabonomic phenotypes for the erlotinib-sensitive and erlotinib-resistant NSCLC cells. The first pair were the isogenically matched PC9 (erlotinib sensitive) and PC9ER (erlotinib resistant) cells both containing ΔE746-A750 EGFRm with an additional T790M (EGFRm/T790M) mutation in PC9ER cells. The second pair included the H3255 and genetically unrelated erlotinib-resistant H1975 cell lines sharing L858R EGFRm, but with an additional T790M mutation in the H1975. PC9ER and H1975 cells displayed significant resistance to erlotinib as compared with their sensitive counterparts (Supplementary Figure S1A). This resistance was limited to EGFR TKIs as PC9ER and PC9 cells were equally sensitive to conventional chemotherapeutic agents (Supplementary Figure S1B). It has been suggested that the EGFR T790M-mediated TKI resistance is due to increased affinity of the receptor for ATP, which displaces competitive inhibitors such as erlotinib [24]. However, both PC9ER and H1975 showed significant resistance even to the irreversible EGFR inhibitor 324674 compared with PC9 and H3255 cells, respectively (Supplementary Figure S1C). This clearly suggests that other unidentified molecular mechanisms also contribute to T790M-mediated TKI resistance.

**Figure 1:**
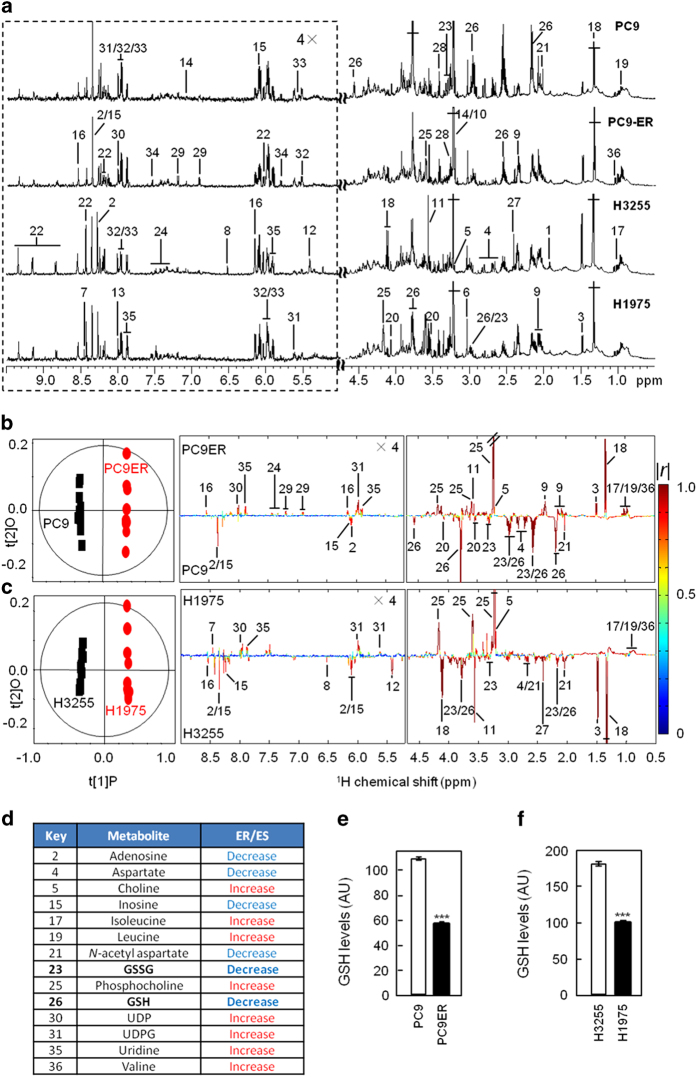
Metabolic characteristics for the erlotinib-resistant and -sensitive cells. (**a**) Typical 600 MHz ^1^H-NMR spectra of aqueous extracts from PC9, PC9ER, H3255 and H1975 cells. The region (*δ* 5.0–9.5) is vertically expanded four times (4×). Data representative of *n*=10. Orthogonal projections to latent structures discriminant analysis (OPLS-DA) score plots (left) and coefficient plots (right) for ^1^H-NMR spectra of aqueous cellular extracts from PC9ER and PC9 showing significantly differentiated metabolites (**b**), H1975 and H3255 (**c**). Models validated by CV-ANOVA, *P*=2.36×10^−17^ (**b**) and *P*=3.04×10^−19^ (**c**). The Q^2^ is 0.99 for both models. The colour scale for coefficient plots reflects the differences in the contribution of metabolite variations between groups. |*r*| cutoff value is 0.602 (*n*=10, *P*<0.05). For identification of peak numbers, see Supplementary Table S1 and d. (**d**) Metabolites showed statistically significant differences between resistant and sensitive cells in both cell line pairs with statistically significant ‘decreases’ or ‘increases’ detected in the erlotinib-resistant (ER) cells as compared with erlotinib-sensitive (ES) ones. (**e**, **f**) GSH levels in PC9 and PC9ER (**e**) or H3255 and H1975 (**f**) cells determined by colorimetric assay. Data are average±s.e.m. of *n*=4. Statistics: Student’s *t*-test. ***P<0.001. See also Supplementary Figures S1 and S2.

To identify these, we comprehensively analysed the ^1^H-NMR metabonomic profiles of our erlotinib-sensitive and -resistant cells. ^1^H-NMR analysis of cell extracts from our cell lines identified 36 metabolites (Figure 1a) for which unambiguous assignments were obtained using various two-dimensional NMR methods (Supplementary Table S1). Statistical analysis of the spectral data by orthogonal projections to latent structures discriminant analysis (OPLS-DA) showed significant metabonomic differences between the erlotinib-resistant and -sensitive cells (Figure 1b and c). Changes in 14 metabolites mainly involved in GSH, amino acids, nucleotides and choline metabolism (Supplementary Figure S2A–C) correlated with resistance in both cell line pairs (Figure 1d; Supplementary Table S2). Noticeably, a significant drop in the intracellular levels of GSH accompanied erlotinib resistance (Figure 1d; Supplementary Table S2). Such GSH decrease observed by NMR was independently confirmed using a colorimetric assay (Figure 1e and f). This was intriguing, as drug resistance was traditionally associated with increased GSH levels [25, 26]. Nevertheless, GSH covalently binds some chemotherapeutic drugs leading to their glutathione-*S*-transferase-mediated extracellular export and resistance of cancer cells to these compounds [27, 28]. Hence, the increased export of this metabolite in complex with erlotinib could account for the lower GSH levels in these resistant cell lines. ^1^H-NMR analysis of the culture medium from our four cell lines disproved this possibility by showing no difference in secreted GSH between TKI-resistant and -sensitive cells (Supplementary Figure S2D). Hence, decreased intracellular GSH levels in erlotinib-resistant cells are likely due to the changes in GSH metabolism.

### Erlotinib-resistant cells have lower expression of GSH-synthesising enzymes

**Figure 2:**
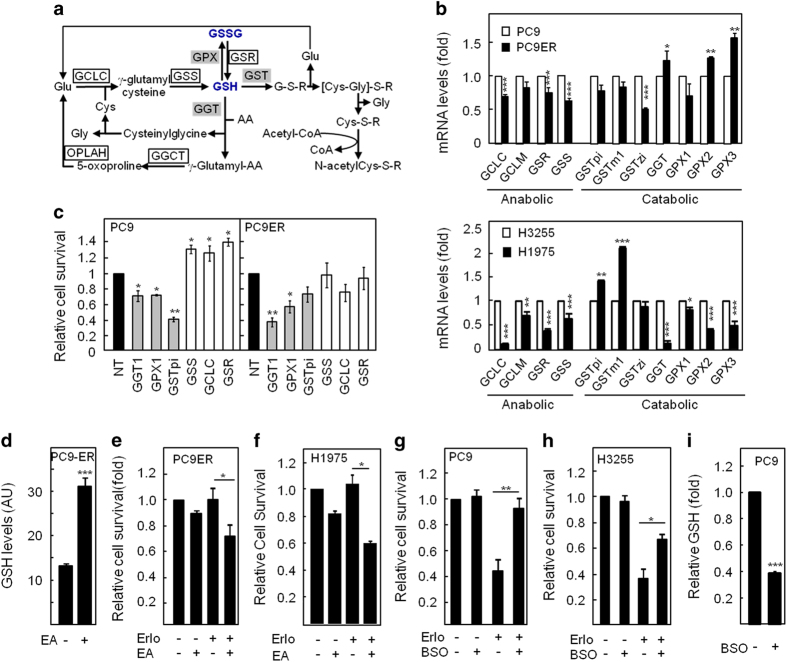
Intracellular GSH levels modulate response to erlotinib. (**a**) Schematics of the GSH metabolic pathway. White boxes, synthesising enzymes; and grey boxes, catabolic enzymes. (**b**) Quantitative reverse transcription PCR for GSH pathway enzymes in PC9, PC9ER, H3255 and H1975 cells. Data are relative mRNAs levels in PC9ER (upper panel) and H1975 (lower panel) normalised to those in PC9 and H3255 cells, respectively. (**c**) PC9 and PC9ER cells were transfected with siRNA targeting GSH-catabolic (grey bars) and synthesising (white bars) enzymes or a non-targeting control (NT) and cell survival to erlotinib (50 nm) monitored by crystal violet staining. Data for the relative survival to erlotinib are normalised to non-targeting control. Survival to erlotinib of PC9ER (**e**) and H1975 (**f**) cells treated with ethacrynic acid (EA) or PC9 (**g**) and H3255 (**h**) cells treated with buthionine sulphoximine (BSO) was monitored by crystal violet staining. Accompanying changes in GSH levels in PC9ER (**d**) and PC9 (**i**) cells were assessed by colorimetric assay. (**e**–**h**) Data are the relative responsiveness to erlotinib normalised to vehicle (−; DMSO). (**b**–**i**) Data representative of ⩾3 experiments and are average of *n*=3±s.e.m. Statistics: (**e**–**h**) analysis of variance, (**b**–**d**, **i**) Student’s *t*-test, *P⩽0.05, **P⩽0.01, ***P⩽0.001. See also Supplementary Figures S3 and S4.

We investigated whether erlotinib-resistant cells differed from their sensitive counterparts in their GSH-metabolic enzymes expression pattern. Quantitative PCR analysis revealed lower messenger RNA (mRNA) levels for GSH-synthesising enzymes (GCLC, GSS and GSR) in erlotinib-resistant cells compared with sensitive ones (Figure 2a and b). In addition, mRNA levels for GCLM, the modulatory subunit of GCLC, were significantly lower in H1975 than in H3255 cells. In contrast, changes in the levels for GSH-catabolic enzymes (GPX1/2/3, GGT and GSTpi/m1/zi) varied greatly between cell line pairs and enzyme subtypes indicating no clear pattern (Figure 2b). Therefore, a reduction in GSH biosynthesis becomes a sound explanation for the decreased GSH levels in EGFRm/T790M erlotinib-resistant cells.

### Targeting GSH metabolism modulates the cellular response to erlotinib

NMR results suggested that lower GSH levels associated with erlotinib resistance. To strengthen this link, we employed small interfering RNAs (siRNAs) for GSH-metabolic enzymes to modulate GSH levels in our cell lines. Silencing of GSH-catabolic enzymes (GGT1, GPX1 and GSTpi) increased the response to erlotinib in both the EGFRm PC9 and EGFRm/T790M PC9ER and H1975 cells (Figure 2c; Supplementary Figure S3C). This correlated with efficient targets’ downregulation and a corresponding increase in GSH levels (Supplementary Figure S3A and B). Conversely, silencing GSH-synthesising enzymes (GCLC, GSS and GSR) lowered cellular GSH levels (Supplementary Figure S3A and B) and rendered the sensitive PC9 cells erlotinib-resistant (Figure 2c).

To validate our siRNA data, we used small-molecule inhibitors targeting the activity of GSH pathway enzymes. Treatment with EA, a known GST inhibitor, increased GSH levels in erlotinib-resistant cells (Figure 2d) causing re-sensitisation of PC9ER and H1975 cells to erlotinib (Figure 2e and f). Similarly, GPXs inhibition using mercaptosuccinate increased intracellular GSH levels (Supplementary Figure S3D) and the response of H1975 cells to erlotinib (Supplementary Figure S3E). Conversely, GCLC inhibition using buthionine sulphoximine in sensitive cells made them erlotinib resistant (Figure 2g and h), an effect associated with decreased GSH levels (Figure 2i). Furthermore, EA was also able to sensitise PC9ER cells to gefitinib by increasing intracellular GSH (Supplementary Figure S4A–C). Taken together, these data suggest that manipulating GSH levels controls the responsiveness of our cell lines to erlotinib.

### The NRF2 pathway controls GSH synthesis and responsiveness to erlotinib

**Figure 3:**
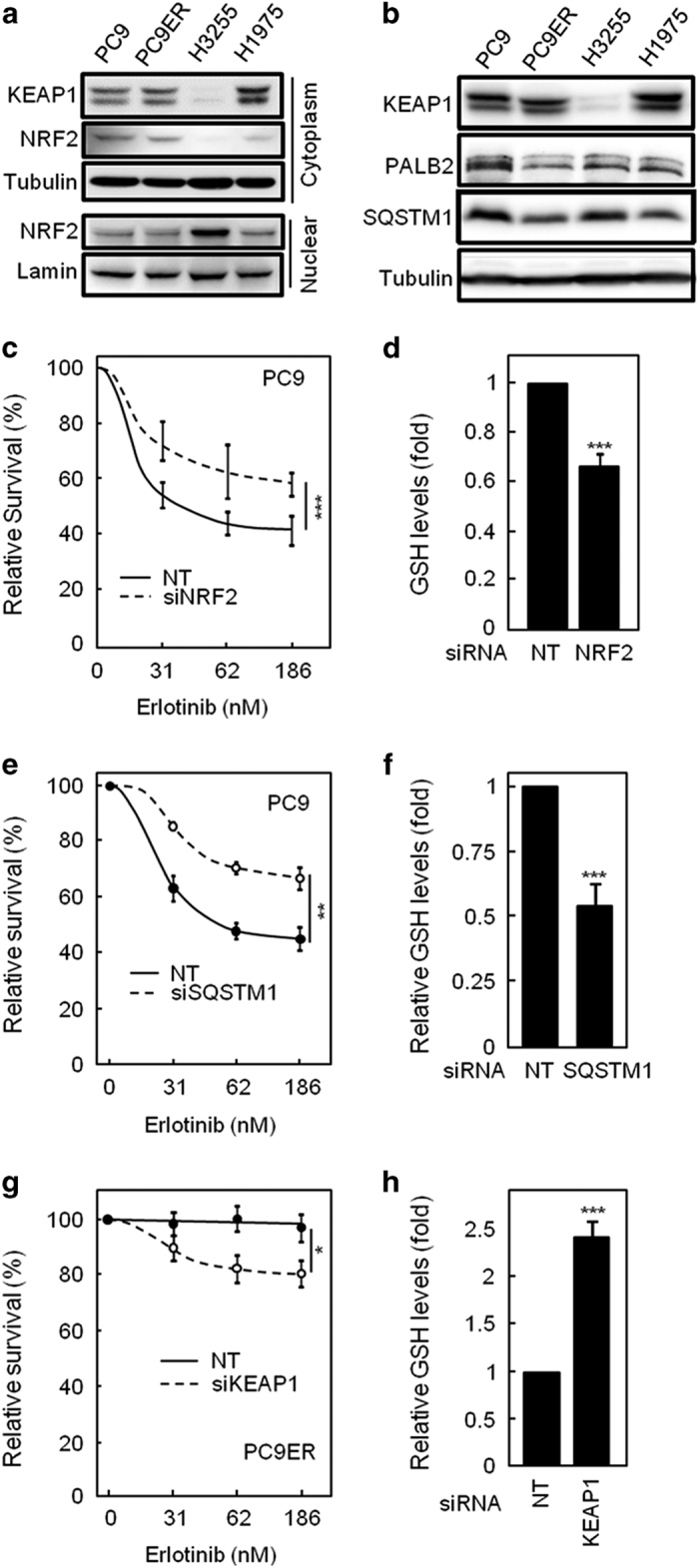
Erlotinib resistance correlates with decreased NRF2 activity. (**a**, **b**) Subcellular fractions (**a**) and total lysates (**b**) from PC9, PC9ER, H3255 and H1975 cells were analysed by SDS-PAGE/western blotting for the indicated proteins. Detection of lamin and tubulin was used as loading controls for nuclear fractions and total lysates or cytoplasmic fractions, respectively. (**c**–**h**) PC9 cells transfected with non-targeting (NT), NRF2 or SQSTM1 siRNAs (**c**–**f**) or PC9ER cells transfected with KEAP1 or NT siRNAs (**g**, **h**) were treated with erlotinib and survival assessed by crystal violet staining (**c**, **e**, **g**). GSH levels were measured by colorimetric assay (**d**, **f**, **h**). (**c**–**h**) Data are average of *n*=4±s.e.m. Statistics: Student’s *t*-test,*P⩽0.05, **P⩽0.01, ***P⩽0.001. See also Supplementary Figures S5 and S6.

GCLC, GSS and GSR are transcriptional targets of NFE2-related factor 2 (NRF2) [29–31], a downstream target of EGFR [32]. We therefore hypothesised that NRF2 activity might be impaired in EGFRm/T790M cells. NRF2’s transcriptional activity requires its nuclear localisation and NRF2 is also degraded through binding to KEAP1, a process counteracted by competitive interaction of the latter protein with PALB2 and/or SQSTM1. Analysis of nucleocytoplasmic fractions and total lysates from our four cell lines revealed that NRF2 or KEAP1 localisation/expression had no differences between PC9 and PC9ER cells, whereas H3255 cells showed higher level of nuclear NRF2 than H1975 cells (Figure 3a; Supplementary Figure S5A). This correlated with increased KEAP1 expression in H1975 as compared with H3255 cells (Figure 3a; Supplementary Figure S5B). Although these results alone may explain the difference in GSH pathway enzymes expression between the latter two cell lines, they cannot account for that seen between PC9 and PC9ER cells. However, SQSTM1 was downregulated in both PC9ER and H1975 cells as compared with their erlotinib-sensitive counterparts (Figure 3b; Supplementary Figure S5C), whereas PALB2 levels were lower in PC9ER as compared with PC9 cells (Figure 3b; Supplementary Figure S5D). Furthermore, NRF2 has been shown to be a transcriptional regulator of SQSTM1 and, indeed, mRNA levels of SQSTM1 were found to be significantly lower in both the resistant cell line pair (Supplementary Figure S5E and F). Hence, inhibition of NRF2 activity through various mechanisms may be linked to erlotinib resistance in NSCLC cells.

To test this hypothesis, we silenced NRF2, SQSTM1, PALB2 and KEAP1 in our cells. siRNA-mediated silencing of NRF2 (Supplementary Figure S6A) rendered PC9 cells erlotinib resistant, a change associated with lower intracellular GSH (Figure 3c and d). Indeed, NRF2-silenced cells showed downregulation of the GSH-synthesising enzymes GCLC and GSR (Supplementary Figure S6B), demonstrating a direct link between NRF2 activity and GSH synthesis. Similarly, SQSTM1 silencing (Supplementary Figure S6C and D) decreased the sensitivity of PC9 cells to erlotinib (Figure 3e) in association with a drop in GSH levels (Figure 3f). Conversely, KEAP1 downregulation (Supplementary Figure S6E) sensitised EGFRm/T790M PC9ER cells to erlotinib (Figure 3g), accompanied by the increased GSH levels (Figure 3h) and increased transcription of GSH-synthesising enzymes GCLC, GSR and GSS (Supplementary Figure S6F). Finally, despite the changes in PALB2 between PC9ER and PC9 cells (Figure 3b), silencing this protein in PC9 cells failed to induce erlotinib resistance or alter GSH levels (Supplementary Figure S7A and B). Hence, the modulation of NRF2 activity through KEAP1 and SQSTM1 regulates the sensitivity of NSCLC cells to erlotinib.

### Inhibition of NRF2 activity and decreased GSH levels are direct consequences of the T790M mutation

**Figure 4:**
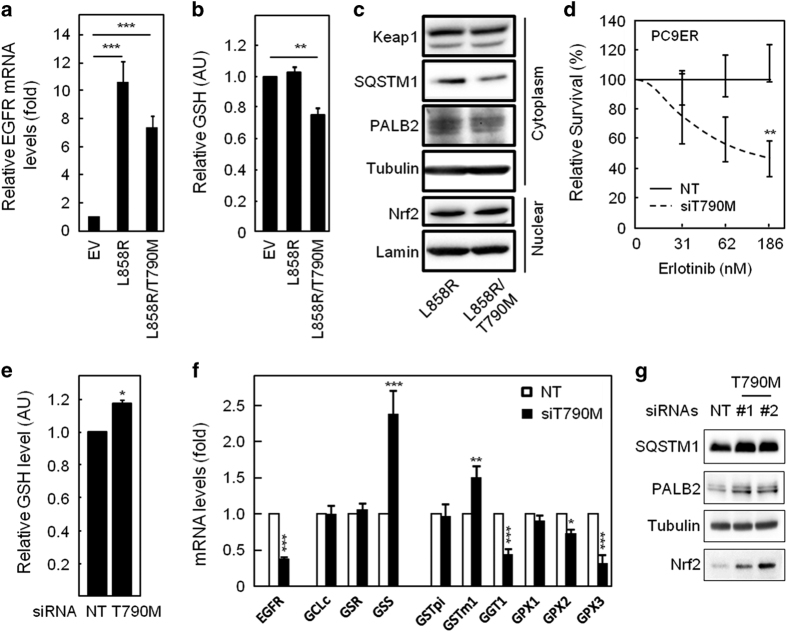
Expression of EGFRm/T790M decreases intracellular GSH levels and NRF2 activity. (**a**–**d**) HEK293 cells were transfected with empty vector control (EV), activated L858R-EGFR or activated/resistant L858R/T790M EGFR-mutant constructs. (**a**) Quantitative reverse transcription PCR for EGFR, (**b**) colorimetric assay for GSH levels and (**c**) cell fractionation followed by SDS-PAGE/western blotting for the indicated proteins were done on stable cell lines. Detection of lamin and tubulin was used as loading controls for nuclear and cytoplasmic fractions, respectively. (**d**–**g**) PC9ER cells transfected with an EGFR T790M-specific or NT siRNAs were subjected to (**d**) treatment with erlotinib before crystal violet staining, (**e**) colorimetric assay for intracellular GSH levels, (**f**) quantitative PCR for GSH metabolic enzymes or (**g**) SDS-PAGE/western blotting. All data representative of ≥3 experiments. (**a**,**b**,**d**–**f**) Values are average of *n*=4±s.e.m. Statistics: (**a**,**b**) analysis of variance, (**d**,**e**,**f**) Student’s *t*-test, *P⩽0.05, **P⩽0.01, ***P⩽0.001. See also Supplementary Figures S6 and S7.

Although lower GSH levels and NRF2 activity were associated with T790M-driven erlotinib resistance in our cell lines, this may still be incidental unless the T790M mutation directly induces these changes. We further expressed the active (L858R) or active/resistant (L858R/T790M) EGFR mutants in HEK293 cells that contain low endogenous EGFR levels (Figure 4a). Unlike expression of the L858R-EGFR, expression of the L858R/T790M double-mutant receptor reduced intracellular GSH levels (Figure 4b). This was associated with reduced PALB2 and SQSTM1 expression (Figure 4c). Conversely, transfection with two independent siRNA sequences previously shown to selectively target T790M-mutant EGFR [33]-sensitised PC9ER cells to erlotinib (Figure 4d; Supplementary Figure S7C and D) and increased GSH levels (Figure 4e). The latter correlated with a reversal of changes in the expression pattern of GSH metabolic enzymes observed between PC9 and PC9ER cells (Figure 4f vs Figure 2b) and with increased PALB2, SQSTM1 and NRF2 levels in T790M-silenced cells (Figure 4g). Therefore, lower GSH levels in T790M NSCLC cells are a direct consequence of acquiring this mutation and the accompanying impairment of NRF2 activity.

### Decrease in GSH correlates with increased nitric oxide levels

**Figure 5:**
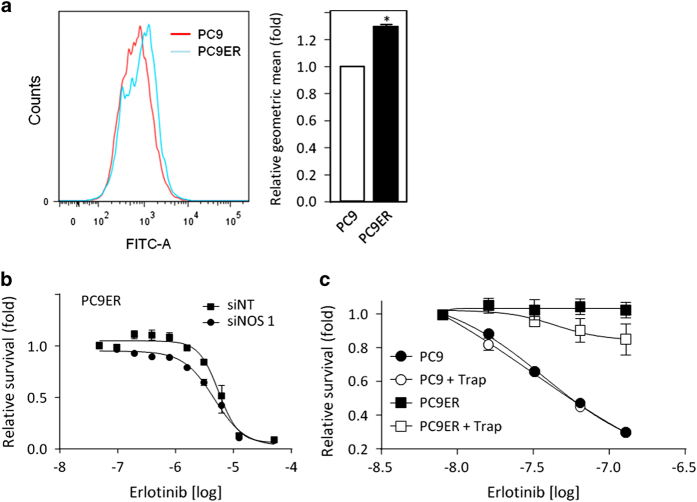
Changes in NO levels modulate erlotinib response. (**a**) NO levels in PC9 and PC9ER cells were compared by fluorescence-activated cell sorting (FACS) using DAF-FM. Left: FACS profile; right: fold changes in geometric mean. (**b**) PC9ER cells transfected with non-targeting (NT) or NOS1 siRNAs or (**c**) PC9 and PC9ER cells treated±an NO-trap were exposed to a dose range of erlotinib. Cell survival was determined by crystal violet staining. Statistics: Student’s *t*-test, *P⩽0.05.

As GSH buffers reactive oxidative species, we investigated whether lower GSH levels in erlotinib-resistant cells associated with elevated reactive oxidative species. We performed flow cytometry analysis in the presence of dihydroethidine and DAF-FM (4-amino-5-methylamino-2′,7′-difluorofluorescein diacetate) to detect superoxide and nitric oxide (NO) species, respectively. Erlotinib-resistant cells showed an increase in NO species (Figure 5a), although they did not show increased superoxide levels. To assess whether this could influence erlotinib resistance, we first silenced the expression of the three NO synthases, NOS1–3. Although siRNA-mediated downregulation of NOS2 and 3 did not impact on erlotinib resistance (not shown), NOS1 silencing sensitised PC9ER cells to erlotinib (Figure 5b). Next, we quenched cellular NO in erlotinib-resistant cells with the NO-trap carboxy-PTIO and revealed that this partially re-sensitised PC9ER cells to erlotinib (Figure 5c). Although these data suggest a role for NO in erlotinib resistance, the levels of changes observed as compared with those seen earlier (Figures 2 and 3) suggest that changes in NO are not solely responsible for resistance downstream of decreased GSH levels.

### EA administration re-sensitises EGFRm/T790M tumours to erlotinib in mouse xenografts

**Figure 6:**
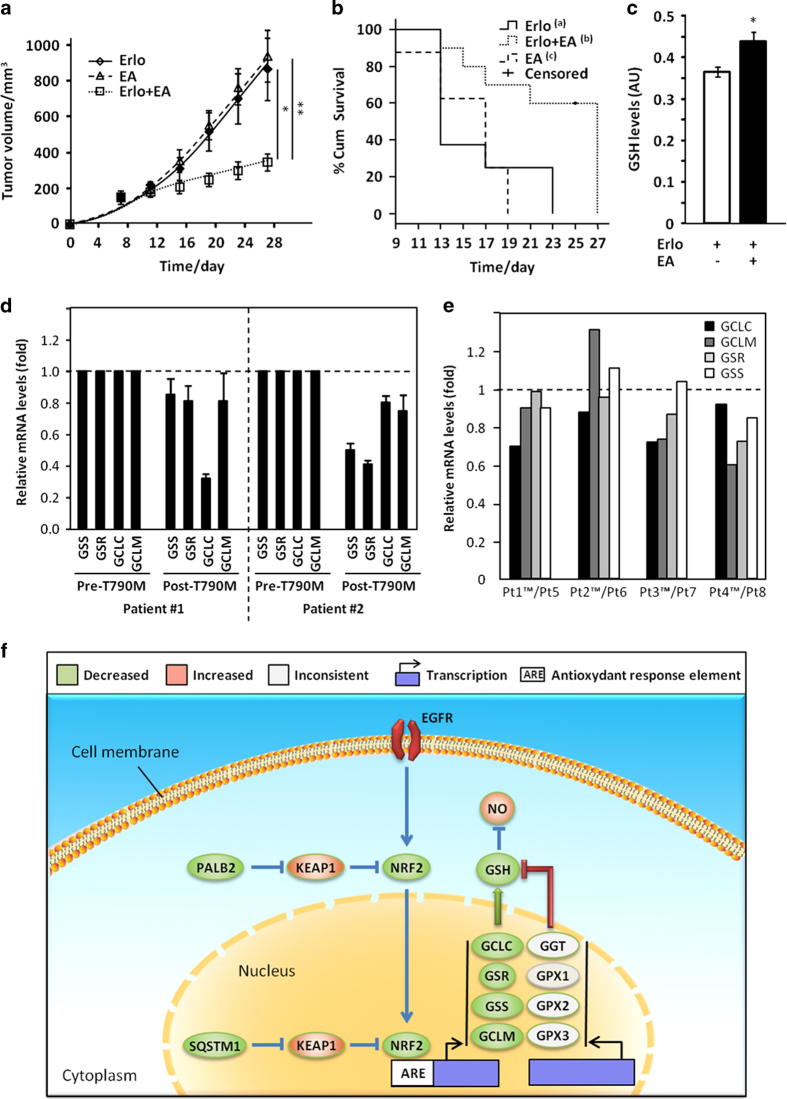
Systemic EA administration re-sensitises PC9ER mouse xenografts to erlotinib. Nude mice (*n*=10/condition) were injected subcutaneously with PC9ER cells and treatment started when tumours reached 100 mm^3^. (**a**) Tumour volume and (**b**) animals survival were monitored for 27 days. (**a**) Data are average±s.e.m. (**b**) End-point events occur when tumour volumes ⩾300 mm^3^. Log-rank test, *P*_ab_<0.01, *P*_bc_<0.01. (**c**) Following the last treatment, intratumoral GSH levels were measured *ex vivo* by colorimetric assay. Statistics: (**a**) analysis of variance, (**c**) Student’s *t*-test, *P<0.05; **P<0.01. GSH-synthesising enzymes expression is decreased in EGFRm/T790M patient tumours. mRNA levels for the indicated enzymes were compared by quantitative PCR in two patients before (pre-T790M) and after (post-T790M) the onset of T790M-mediated erlotinib resistance (**d**) or by RNA-Seq in four pairs of unrelated patients with (Pt1-4) or without (Pt5-8) T790M (**e**). Data in T790M samples are normalised to those in the corresponding non-T790M samples. (**f**) Model of changes occurring downstream of T790M EGFR.

The GST inhibitor EA restored GSH levels and erlotinib sensitivity in EGFRm/T790M cells *in vitro* (Figure 2). EA is still used as a diuretic in humans for conditions including high blood pressure and heart failure [34]. Hence, we hypothesised that co-administration of physiologically relevant doses of EA might improve the responsiveness of EGFRm/T790M tumours to erlotinib *in vivo*. PC9 or PC9ER cells were injected subcutaneously in *nude* mice and tumours were left to grow to 100 mm^3^. The animals were then treated daily with erlotinib and EA alone or in combination. Co-administration of the drugs greatly inhibited tumour growth with 60% of the animals showing tumour volumes ⩽300 mm^3^ at 25 days, whereas those treated with either drug alone showed more extensive disease (Figure 6a). This was associated with increased survival (Figure 6b) and intra-tumoural GSH levels in combination-treated animals (Figure 6c). EA did not have any effect on erlotinib sensitivity of PC9 xenografts in agreement with the lack of further added sensitisation to erlotinib obtained with this inhibitor *in vitro* (Supplementary Figure S7E). Thus, co-administration of EA is probably a viable strategy for the management of erlotinib-resistant cancers in humans.

### Decreased GSH synthetic enzymes expression characterises erlotinib-resistant patients

Finally, we assessed whether the decrease in GSH-synthetising enzymes observed in EGFRm/T790M cell lines *in vitro* also occurred in patients. First, we performed quantitative PCR for GSS, GSR, GCLC and GCLM in paired biopsy samples from two patients before (EGFRm alone) and after acquiring EGFRm/T790M-mediated erlotinib resistance. In both cases, resistance was accompanied by a decrease in one or both of the rate-limiting enzymes for GSH biosynthesis, GCLC and GSS (Figure 6d). Moreover, this association was not limited to syngeneic samples, as RNA-Seq of four pairs of unrelated patients’ biopsies revealed lower expression of at least one of these enzymes in T790M tumours as compared with non-T790M samples (Figure 6e). Therefore, decreased expression of GSH synthetic enzymes is probably associated with T790M-mediated erlotinib resistance in lung cancer patients.

## Discussion

EGFR TKIs such as erlotinib offer therapeutic benefit to NSCLC patients harbouring EGFRm [1–3]. However, the rapid development of resistance due in 50% of cases to acquisition of the secondary T790M EGFR mutation greatly limits the ability of these agents to prolong patient survival [5–8]. Although decreased affinity of the EGFRm/T790M for erlotinib was thought responsible and new irreversible inhibitors may be promising in circumventing this, additional mechanisms of resistance are likely to be present. Indeed, EGFRm/T790M cells still demonstrate significant loss of sensitivity to an irreversible compound (Supplementary Figure S1C). This suggested that resistance to erlotinib in EGFRm/T790M NSCLC cells is mediated through additional mechanisms.

Accumulating evidence suggests EGFR mutations to drive alteration in metabolic signatures, however, majority of them fail to demonstrate efficacy of targeting these molecules in clinical settings or *in vivo* models [35–37]. To identify novel resistance pathways, we performed ^1^H-NMR metabonomic analysis of two independent NSCLC erlotinib-sensitive/resistant cell line pairs (PC9/PC9ER and H3255/H1975 cell lines). These were chosen according to the several criteria. First, both resistant cell lines shared the same T790M resistance mutation. Second, although PC9ER cells were obtained through selecting PC9 cells with erlotinib making these two lines relatively isogenic, H3255 and H1975 cells are genetically unrelated. Third, the primary EGFR-activating mutations in the two cell line pairs were different (ΔE746-A750 for PC9/PC9ER cells, L858R for H3255/H1975 cells). These criteria maximised the opportunity for metabolic changes shared by both cell line pairs to be solely dependent on the T790M mutation. One of the most striking differences highlighted by our analysis was a decrease in GSH levels in erlotinib-resistant cells (Figure 1). The GSH pathway has long been involved in cancer drug resistance [27, 28]. However, this was traditionally associated with increased GSH levels [25, 26]. Indeed, GSH covalently binds to some drug molecules in a GST-dependent manner leading to their cellular export and quenches reactive oxidative species often requiring for these compounds to act [27, 28]. Therefore, an association between decreased GSH levels and EGFR TKI resistance was surprising and warranted further investigation of its relevance to erlotinib responses.

Our experiments demonstrated that inhibition of GSH biosynthesis by either RNAi or small molecules made erlotinib-sensitive cells resistant to the drug (Figure 2d–i). Conversely, inhibition of GSH-degradation re-sensitised resistant cells to erlotinib (Figure 2d–f). Hence, changes in GSH levels alone can modulate the response of NSCLC cells to this drug and decreased GSH levels accounts for erlotinib resistance in PC9ER and H1975 cells. Comparative analysis revealed a transcriptional downregulation of GSH-synthesising enzymes in T790M cells (Figure 2b) due to the impairment of NRF2, a downstream mediator of EGFR responsible for transcription of these enzymes (Figure 3a and b). This occurred via upregulation of the NRF2 inhibitor KEAP1 and/or downregulation of PALB2 and SQSTM1, two proteins involved NRF2 stabilisation. Indeed, siRNA-mediated silencing of KEAP1 in T790M cells sensitised them to erlotinib (Figure 3g and h), whereas that of SQSTM1 or NRF2 made sensitive cells resistant to this drug (Figure 3c–f). Importantly, decreased NRF2 activity and GSH levels in resistant cells were a direct consequence of acquiring the T790M mutation as introducing EGFRm/T790M in HEK293 cells, rather than EGFRm alone, reproduced the changes associated with erlotinib resistance (Figure 4a–c). Conversely, silencing EGFRm/T790M in PC9ER cells reverted the changes in GSH levels and metabolic enzymes seen upon acquisition of resistance by PC9 cells (Figure 4d–g).

It is unclear by what mechanism(s) the T790M mutation induces the observed transcriptional changes as the higher kinase activity of EGFRm/T790M [9] should further enhance NRF2 activity. However, mutant EGFRs differ from their wild-type counterparts in their subcellular localisation [38], which probably results in the EGFRm/T790M having different signalling partners as EGFRm or wild-type EGFR. Further research will be required to investigate this possibility.

We next attempted to identify the mechanism by which decreased GSH levels cause erlotinib resistance. GSH is a major cellular antioxidant [39], and its reduced expression could result in increased reactive oxidative species. In addition, single-nucleotide polymorphisms in antioxidant genes have been demonstrated to be associated with survival outcome in patients receiving TKI therapy [40]. Although superoxide levels were unchanged, NO levels were raised in PC9ER as compared with PC9 cells (Figure 5a) and NOS1 silencing or NO quenching sensitised PC9ER cells to erlotinib (Figure 5b and c). GSH is known to neutralise NO and protect against protein nitrosylation [41, 42]. It is worth noting that EGFR is a target of *S*-nitrosylation [43], but the consequence of this on erlotinib response is currently unknown. However, although our data suggest that NO probably contributes to erlotinib resistance, this does not fully explain the effects of reduced GSH. Glutathionylation has a role in disease state by modifying the function of target proteins [44] and assessing changes to the glutathionylation profile may identify proteins involved in EGFRm/T790M-mediated erlotinib resistance.

Regardless of the mechanism underlying erlotinib resistance downstream of decreased GSH levels, we showed that the GSH pathway could be manipulated for therapeutic benefit. Indeed, systemic administration of clinically relevant doses of EA, a GST inhibitor [45], increased the intra-tumoural GSH levels (Figure 6c) and re-sensitised EGFRm/T790M tumours to erlotinib in a cancer cell xenograft mice model (Figure 6a–c). As EA is an orally available diuretic used in humans with limited toxicity [34], our findings could rapidly translate into clinical practice if this sensitisation also occurs in humans. Moreover, EA has already been used together with classical chemotherapeutics such as alkylating agents to prevent their GST-mediated cellular export [34], leading to improved clinical outcome. Therefore, EA may help manage erlotinib resistance in EGFRm-NSCLC patients and improve response to follow-on chemotherapeutic regimen. However, it is unclear whether decreased GSH levels only occurs downstream of the EGFRm/T790M or if this is a common feature of other erlotinib resistance pathways such as c-Met amplification. Answering this before clinical exploitation of our findings will help more accurate patient selection for EA/erlotinib combined trials.

Finally, we show our findings to be clinically relevant using EGFRm and EGFRm/T790M lung cancer samples (Figure 6d and e). The reduced number of samples analysed reflects the fact that repeated biopsy in NSCLC following the onset of EGFR TKI resistance is rare, although this practice is now changing. Nevertheless, we demonstrate in both syngenic and unrelated patient samples that mRNA levels for GSH-synthesising enzymes are decreased in T790M tumours. Hence, probing for GSH-synthesising enzymes may help, in a recurrent setting, to predict the response to combinatorial therapies of erlotinib and GSH level increasing agents.

To sum up, we demonstrate that decreased intracellular level of GSH could mediate T790M-driven erlotinib resistance in NSCLC and highlight the molecular events involved (Figure 6f). Therapeutic strategies that increase intra-tumoural GSH levels may revert erlotinib resistance in the clinic.

## Materials and Methods

### Materials

Mercaptosuccinic acid (used at 50 μm), buthionine sulfoximine (used at 40 μm) and EA (used at 50 and 100 μm in PC9 and H1975 cells, respectively) were purchased from Sigma (St Louis, MO, USA), whereas EGFR Inhibitor 324674 was from Santa Cruz (Dallas, TX, USA) and Merck (Kenilworth, NJ, USA), respectively. Antibodies against GSTpi, GPX1, GSS, GSR, GCLC, GSTpi, SQSTM1 and DPP3 were from Abcam (Cambridge, UK); antibodies targeting NRF2 and KEAP1 were from Santa Cruz and anti-PALB2 was from Novus (Abingdon, UK). The specificity of all antibodies employed here was assessed by disappearance of the respective signal following selective targeting of the expression of the corresponding protein by siRNA treatment. Quantitect primers targeting GSTpi, GPX1, GPX1, GSS, GSR and GCLC were from Qiagen (Valencia, CA, USA). All other primers were synthesised by Sigma. SiRNAs were purchased from Dharmacon (Little Chalfont, UK). Dihydroethidine was from Invitrogen (Waltham, MA, USA) and DAF-FM from Sigma.

### Cell culture

All cell lines were obtained from the CRUK cell line bank where they were authenticated and mycoplasma status assessed through regular testing in our lab. Cell lines were grown in Roswell Park Memorial Institute with 10% fetal bovine serum at 37 °C, 5% CO_2_.

### Extraction of the intracellular metabolites

Intracellular metabolites were extracted as reported previously [46, 47] with some modifications. In brief, 10^7^ cells per condition were trypsinised and washed thrice in ice-cold phosphate-buffered saline before metabolite extraction. Cell pellets were resuspended in 0.6 ml cold water/methanol (1:2) and subjected to three freeze–thaw cycles before sonication in a wet ice bath for 15 min (cycles: 1 min pulse followed by 1 min pause). Samples were then centrifuged (3200 *g*/4 °C, 10 min) and supernatants transferred into cold Eppendorfs. The remaining pellets were extracted twice more by the same method. Supernatants from the three subsequent extractions were combined, centrifuged (12 000 *g/*4 °C, 10 min) and freeze-dried following vacuum-driven methanol evaporation. Lyophilised samples were stored at −80 °C. Ten biological replicates were used for each group of cells.

### Cellular metabonomic analysis by ^1^H-NMR

Freeze-dried intracellular metabolites extracts were dissolved in 600 μl phosphate buffer 0.1 m (pH 7.4, 99.9% D_2_O) containing 0.001% sodium 3-trimethylsilyl-1-[2,2,3,3-^2^H4] propionate as previously described [48]. All samples were centrifuged (12 000 *g*/4 °C, 10 min) after short vortexing and supernatants transferred into the 5 mm NMR tubes for NMR detection. All one-dimensional ^1^H-NMR spectra were acquired on a Bruker AVIII 600 MHz NMR spectrometer equipped with a cryogenic probe (BrukerBiospin, Rheinstetten, Germany) at 298 K. The first increment of NOESY pulse sequence was employed with continuous wave irradiation on the water peak during recycle delay and mixing time for water suppression. Recycle delay of 2 s and mixing time of 100 ms were set. The 90° pulse was adjusted to 10 μs approximately and 64 scans were collected into 32 k data points with the spectral width of 20 p.p.m. For metabolite assignments, two-dimensional NMR spectra including ^1^H–^1^H COSY, ^1^H–^1^H TOCSY, ^1^H *J*-resolved, ^1^H–^13^C HSQC and ^1^H–^13^C HMBC for typical samples were acquired and processed as described previously [49].

### NMR data analysis

The spectral region at *δ* 0.5–9.5 was integrated into bins with the width of 0.002 p.p.m. (1.2 Hz) using AMIX package (v3.8, BrukerBiospin). The range (*δ* 4.7–5.2) was removed to eliminate the effects of water peak suppression. Each bin area was normalised to the total area of the respective spectrum. Multivariate data analysis was performed with the software SIMCA-P+ (v 12.0, Umetrics, Umea, Sweden). The model was built using the orthogonal projection to latent structure-discriminant analysis (OPLS-DA) [50] with Pareto variance (Par) scaling and sevenfold cross-validation. The parameter *R*^2^*X* was the variation of *X* explained by the model and *Q*^2^ represented the predictability of the model. The validation of all the models was further ensured by CV-analysis of variance (*P*<0.05) [51]. To assist the biological interpretation of the loadings generated from the models, the loadings was firstly back-transformed [52] and then plotted with colour-coded OPLS-DA coefficients in MATLAB 7.1 using an in-house script [53]. The colour code corresponded to the absolute value of the OPLS-DA coefficients (|*r*|), indicating the contribution of each variable to explain the intergroup differentiation. The value of |*r*|, >0.602, was considered to be significant (*n*=10, *P*<0.05).

### GSH colorimetric assay

A GSH colorimetric assay kit was purchased from BioAssay Systems (Hayward, CA, USA) and used according to the manufacturer’s instructions.

### siRNA transfection

A total of 1×10^4^ PC9, PC9ER or H1975 cells per well in six-well plates were transfected with siRNAs at 25 nm (Dharmacon) for 24 h using RNAiMax (Invitrogen) following the manufacturer’s protocol. Each protein was targeted with a mix of four sequences. A total of 4×10^3^ cells were re-seeded and then incubated at 37 °C/5% CO_2_ for 24 h for target silencing before further experiment steps.

### Cell survival assay

For EA, buthionine sulfoximine and mercaptosuccinic acid, cells were pretreated for 4 h before erlotinib addition (100 nm) for 48 h. Cells were then fixed and stained for 20 min with a 25% methanol/0.5% crystal violet solution. Plates were washed in running water, air-dried and the stain dissolved in 10% acetic acid on a shaker before absorbance at 595 nm.

### Quantitative PCR

Total cellular mRNA was extracted using the RNeasy kit (Qiagen) and converted to complementary DNA with a High Capacity cDNA Reverse Transcription Kit (Applied Biosystems, Waltham, MA, USA). mRNA levels were quantified using a Fast SYBR Green Master Mix (Applied Biosystems) on a 7900HT Fast Real time PCR System (Applied Biosystems). qBase software (San Francisco, CA, USA) was used for data analysis using TATA-binding protein and β-actin as internal controls. The primers used were listed below (F, forward; R, reverse): GCLm: (F): 5′-GGCACAGGTAAAACCAAATAGTAAC-3′, (R): 5′-CAAATTGTTTAGCAAATGCAGTCA-3′; GPX2: (F): 5′-TAAGTGGGCTCAGGCCTCTCT-3′, (R): 5′-GGTCATAGAAGGACTTGGCAATG-3′; GPX3: (F): 5′-GACAAGAGAAGTCGAAGATG-3′, (R): 5′-CTTCCTGTAGTGCATTCAGTT-3′; GSTz1: (F): 5′-TCCTATTTCCGAAGCTCCTGC-3′, (R): 5′-TTCAGTGCCTGGAAGTCCTTAG-3′; GSTm1: (F): 5′-CTATGATGTCCTTGACCTCCACCGTATA-3′, (R): 5′-ATGTTCACGAAGGATAGTGGGTAGCTGA-3′; Beta-Actin: (F): 5′-TCCTCCTGAGCGCAAGTACTC-3′, (R): 5′-CTGCTTGCTGATCCACATCTG-3′; KEAP1: (F): 5′-CAGATTGGCTGTGTGGAGTT-3′, (R): 5′-GCTGTTCGCAGTCGTACTTG-3′; SQSTM1: (F): 5′-CTGGGACTGAGAAGGCTCAC-3′, (R): 5′-GCAGCTGATGGTTTGGAAAT-3′; TBP: (F): 5′-TGCACAGGAGCCAAGAGTGAA-3′, (R): 5′-CACATCACAGCTCCCCACCA-3′; NRF2 primers: (F): 5′-GAGAGCCCAGTCTTCATTGC-3′, (R): 5′-TGCTCAATGTCCTGTTGCAT-3′. Primers against the other targets were purchased from Qiagen: GCLc (QT00037310), GGT1 (QT00029470), GPX1 (QT00203392), GSTp1 (QT00086401), GSS (QT00014413) and GSR (QT00038325).

### Tissue mRNA extraction and quantitative PCR

The origin of tissues and techniques used are as previously reported [54]. In short, samples were obtained from EGFR-mutant lung adenocarcinoma patients with acquired erlotinib resistance under Human Investigations Protocol #111000928 (Yale Cancer Center, New Haven, CT). Those were reviewed by a pathologist to ensure adequate tumour content. Tumour areas were circled and microdissection performed to enrich for tumour content.

### Tissue mRNA extraction and RNA-Seq

The Illumina TruSeq RNA Sample Preparation Kit (Illumina Cambridge Ltd, Essex, UK) was used for RNA tissue extraction and analysis done as previously described [55].

### Western blotting

Cells were lysed using 0.5% Triton X-100, 150 mm NaCl, 2 mm EDTA, 10% glycerol supplemented with protease inhibitors cocktail tablets (Roche Diagnostics, Indianapolis, IN, USA), 10 mm β-glycerophosphate, 1 mm Na_3_VO_4_ and 10 mm NaF. Equal protein amounts were analysed by SDS-PAGE/western blotting using the antibodies indicated.

### Flow cytometry analysis of oxidative species

Cells (15×10^4^/well in a six-well plate) were treated with 10 μm DAF-FM or dihydroethidine for 30 min, washed with phosphate-buffered saline, trypsinised, pelleted and resuspended in 1 ml of phosphate-buffered saline before flow cytometry using a BD FACSCalibur (DB Biosciences, Oxford, UK). The geometric mean intensity was determined using FlowJo (Tree Star Inc, Ashland, OR, USA).

### Animal experiments

A total of 5x10^6^ PC9ER or PC9 cells were injected subcutaneously into the flank of female BALB/c nude mice and the tumours grew until they reached 100 mm^3^. Mice were then randomized into three groups (*n*=10) and treated by intraperitoneal injection of 25 mg kg^−1^ per day erlotinib/0.5% w/v methylcellulose and/or 6 mg kg^−1^ per day EA/1% Tween 80 in distilled water. Such treatments were administered daily from day 7 to 26. Tumours were measured by caliper and volumes calculated as *V*=1/2*L*W^2^ (*L*; length, *W*; width of tumour). Data analysis was performed by an investigator blinded to the experimental conditions. All experiments complied with ethical regulations as enforced by the local committee.
