# Supplementary material for: Decreased glutathione biosynthesis contributes to EGFR T790M-driven erlotinib resistance in non-small cell lung cancer
Source: Cell Discov. 2016 Sep 27;2:16031–. doi: 10.1038/celldisc.2016.31 (PMC5037574; doi:10.1038/celldisc.2016.31)

SUPPLEMENTARY DATA

Decreased glutathione biosynthesis contributes to EGFR T790M-driven erlotinib resistance in non-small cell lung cancer

Hongde Li[‡#], William Stokes[¶#], Emily Chater[¶#], Rajat Roy[¶#], Elza de Bruin[§Π], Yili Hu[‡], Zhigang Liu[‡], Egbert F. Smit[&], Guus J.J.E. Heynen[※], Julian Downward[§], Michael J. Seckl[¶*], Yulan Wang[‡△],Huiru Tang[†‡*], Olivier E. Pardo[¶*]

[†] State Key Laboratory of Genetic Engineering, Ministry of Education Key Laboratory of Contemporary Anthropology, Collaborative Innovation Centre for Genetics and Development, Shanghai International Centre for Molecular Phenomics, Metabonomics and Systems Biology Laboratory, School of Life Sciences, Zhongshan Hospital, Fudan University, Shanghai, 200438, China. [‡]Key Laboratory of Magnetic Resonance in Biological Systems, National Centre for Magnetic Resonance in Wuhan, State Key Laboratory of Magnetic Resonance and Atomic and Molecular Physics, Wuhan Institute of Physics and Mathematics, Chinese Academy of Sciences, Wuhan 430071, China. [¶]Division of Cancer, Department of Surgery and Cancer, Imperial College, Hammersmith Hospital, Du Cane Road, London W12 0NN, UK. [§]Signal Transduction laboratory, CRUK London Research Institute, London, UK. [&]Dept. Pulmonary Diseases, VU University Medical Centre and [※]Section of molecular carcinogenesis, Netherlands Cancer Institute, Amsterdam, The Netherlands. [△]Collaborative Innovation Centre for Diagnosis and Treatment of Infectious Diseases, Hangzhou, China. [Π]AstraZeneca, Personalised Healthcare & Biomarkers, Molecular Diagnostics, Darwin, Building 310, Cambridge Science Park, Milton Road, Cambridge CB4 0WG, UK.

[#]These authors have contributed equally to the work.

[*]**Corresponding authors:** o.pardo@imperial.ac.uk, huiru_tang@fudan.edu.cn, m.seckl@imperial.ac.uk

SUPPLEMENTARY TABLES

Supplementary Table 1: NMR data for metabolites identified and assigned. Keys refer to the peaks labelled on **Figure 1A**. Both the chemical shifts for proton (δ^1H) and carbon ($\delta^{13}C$) NMR are tabulated. [a] Multiplicity: singlet(s), doublet(d), triplet(t), quartet(q), doublet of doublets(dd), double of triplets (dt), multiplet(m). [b] The signals or the multiplicities were not determined.

Supplementary Table 2: Metabolites showed statistically significant differences in both cell line pairs between erlotinib-resistant and erlotinib-sensitive cells. "decrease" or "increase" meant statistically significant such differences were detected in the erlotinib-resistant cells as compared to sensitive ones. Keys refer to the peaks labelled on **Figure 1B and C**. The chemical shifts for the identified metabolites and the OPLS-DA coefficient for both cell line pairs are shown.

SUPPLEMENTARY FIGURES

Supplementary Figure 1: (**A** and **C**) PC9ER and H1975 cells are resistant to the EGFR TKIs. PC9, PC9ER, H3255 and H1975 cells were treated with increasing concentrations of erlotinib (**A**) or 324674 (**C**) for 48 h before crystal violet staining. (**B**) PC9 and PC9ER cells do not display differential sensitivity to classical chemotherapeutic agents. PC9 and PC9ER cells were treated with increasing concentrations of cisplatin, taxol and etoposide for 48 hours prior to crystal violet staining. Results shown are representative of at least three independent experiments. Data are average ± SEM of quadruplicates, ***$P < 0.001$.

Supplementary Figure 2: (A-C) Schematic representation of additional metabolic pathways differentially modulated in erlotinib-resistant and sensitive cells. Red; metabolites with increased and Blue; decreased levels in resistant cells as compared to their sensitive counterparts. **(D)** Representative ^{1}H-NMR spectra from the cell culture media of PC9, PC9ER, H3255 and H1975 cells. Cells were grown for 4 days in complete medium. The media were then collected and analysed by NMR for their GSH content. The spectra are zoomed onto the chemical shifts area corresponding to GSH and GSSG. The top line corresponds to a purchased GSH/GSSG mixture internal control. The spectra shown are representative of 10 replicates per cell lines.

Supplementary Figure 3: qPCR controls for siRNA-mediated silencing of GSH metabolic enzymes and accompanying changes in GSH levels. **(A)** PC9 and PC9ER cells were transfected with siRNAs targeting the indicated enzymes. 48 h later, transfected cells were subjected to RT-qPCR to assess the down-regulation of the target mRNAs. Data are shown are average of quadruplicates ± SEM. **(B)** GSH levels were measured by colorimetric assay in PC9 or PC9ER cells downstream of the silencing of selected targets. Data are shown are representative of at least three experiments. Results are average of quadruplicates ± SEM. (B) Statistical analysis, Student *t*-test with NT taken as reference. *$P < 0.05$, **$P < 0.01$. **(C)** H1975 cells were transfected with siRNAs for the indicated enzymes or a non-targeting sequence (NT) prior to treatment with the IC_{50} concentration of erlotinib for H3255 cells. Cell survival was determined by crystal violet staining and normalised to that of the NT condition. **(D)** Intracellular GSH levels were measured in H1975 cells treated with or without mercaptosuccinate (MS) using a colorimetric assay. **(E)** H1975 cells were incubated in the presence or absence of MS for 2 h prior to treatment with or without erlotinib for 48 h. Cell

viability was assessed by crystal violet staining. Results shown are representative of experiments performed at least three times. Data are average of quadruplicates ± SEM. Statistical analysis: (C-D) *t*-test, (E) ANOVA. ***$P < 0.001$, **$P < 0.01$, *$P < 0.05$.

Supplementary Figure 4: PC9ER cells treated with or without Ethacrynic Acid (EA) were exposed to varying doses of Gefitinib for 48 hours and cell survival monitored by crystal violet staining (**A**). Fold change in cell survival at the IC50 dose was determined and data normalised to the cell viability with Gefitinib alone (**B)**. Fold change in GSH was measured in cells treated with or without Gefitinib, EA or combination (**C**). ***$P < 0.001$, **$P < 0.01$.

Supplementary Figure 5: Three replicates for the Western blots shown in Fig 4A and B were quantified using the optical densitometry function in ImageJ and the results obtained for **(A)** NRF2, **(B)** KEAP1, **(C)** SQSTM1 and **(D)** PALB2 normalised to the corresponding control cell line and plotted. (**E-F**) mRNA level of SQSTM1 was quantified for 4 cell lines and results were normalised to the corresponding control cell line. Results shown are representative of experiments performed at least three times. Data are average of triplicates ± SEM. For PC9ER, PC9 cells were used as control while for H1975, H3255 were used for normalisation. Statistical analysis: *t*-test. ***$P < 0.001$, **$P < 0.01$,*$P < 0.05$.

Supplementary Figure 6: (**A, C and E**) qPCR controls for silencing of NRF2, SQSTM1 and KEAP1 in the indicated cell lines. (**B, D and F**) Accompanying changes in the mRNA levels for GSH synthesising enzymes as determined by qPCR. Data shown are representative of at least three independent experiments. Results are average of quadruplicates ± SEM. Statistical analysis: *t*-test, *$P < 0.05$, ***$P < 0.001$.

Supplementary Figure 7: Silencing of PALB2 in PC9 cells does not modulate their sensitivity to erlotinib. (**A and B**) Cells were transfected with siRNA targeting PALB2 or with a non-targeting control for 48 h prior to exposure to a dose range of erlotinib for 2 days (A) or GSH levels measurements using a colorimetric assay (B). Cell survival was assessed using crystal violet staining. Results shown are representative of experiments performed in triplicate. Data are average of quadruplicates ± SEM.

Silencing of T790M-EGFR using selective siRNA does only modify EGFR expression in T790M-EGFR containing cells as assessed by qPCR. PC9 and PC9ER cells were transfected with non-targeting (NT) or two separate T790M-targeting siRNAs (**C and D**) and subjected to qPCR for EGFR using primers detecting equally T790M and non-T790M EGFRs. Results shown are normalised to the corresponding NT condition. Statistical analysis: Student *t*-test, **$P < 0.01$, ***$P < 0.001$.

(E) Response to erlotinib during systemic EA administration in PC9 mouse xenografts. Nude mice (n=10/condition) were injected subcutaneously with PC9 cells and treatment started when tumours reached 100 mm^3. Tumour volume was monitored for 2 weeks**.**

Supplementary Table 1: NMR data for metabolites identified and assigned. Keys refer to the peaks labelled on **Figure 1A**. Both the chemical shifts for proton (δ^1H) and carbon (δ^{13}C) NMR are tabulated. [a] Multiplicity: singlet(s), doublet(d), triplet(t), quartet(q), doublet of doublets(dd), double of triplets (dt), multiplet(m). [b] The signals or the multiplicities were not determined.

Key	Metabolites	Assignment	δ^1H(multiplicity[a])	δ^{13}C
**1**	Acetate	CH_3	1.92(s)	26.1
		COO^-		184.2
**2**	Adenosine	CH-ring	8.34(s)	143.2
		CH-ring	8.178(s)	155.4
		C1H-ribose	6.08(d)	89.5
		C2H-ribose	4.77	76.7
		C4H-ribose	4.28	88.4
		C3H-ribose	4.42	73
		C5H-ribose	3.85/3.9	64.1
**3**	Alanine (Ala)	α-CH	3.78(q)	53.1
		β-CH_3	1.48(d)	18.9
		COO(H)		178.5
**4**	Aspartate (Asp)	α-CH	3.90(m)	54.8
		β-CH	2.69(dd)	39.2
		β'-CH	2.81(dd)	
		γCOO(H)		180.6
		COO(H)		177.4
**5**	Choline	α-CH_2	3.99	70.0
		β-CH_2	3.56	58.6
		N-CH_3	3.20(s)	56.6
**6**	Creatine	CH_3	3.04(s)	39.6
		CH_2	3.93(s)	56.5
		N=C		159.6
		COO(H)		177.3
**7**	Formate	H COO^-	8.46(s)	b
**8**	Fumarate	C2,3H	6.52(s)	138.2
		COO^-		b
**9**	Glutamate (Glu)	α-CH	3.76(t)	57.3
		β-CH_2	2.08(m)	29.8
		γ-CH_2	2.35(m)	36.2
		δCO		183.9
		COO(H)		177.4
**10**	Glycerophosphocholine(GPC)	1-CH_2	3.60(dd)	b
		2-CH	3.89(m)	b
		3-CH_2	3.72(dd)	b
		α-CH_2	4.32(t)	b
		β-CH_2	3.68(t)	b
		N-CH_3	3.23(s)	56.7
**11**	Glycine (Gly)	α-CH_2	3.56(s)	44.1
		COO(H)		175.1
**12**	Glycogen		5.41(broad peak)	102.4
			3.97	b
			3.85	b

			3.7	b
**13**	Guanosine	CH-ring	8.0(s)	140.9
		C1H-ribose	5.92(d)	91.7
		C3H-ribose	4.41(dd)	73.4
		C4H-ribose	4.24(dt)	88.21
		C5H-ribose	3.84	64
**14**	Histidine (His)	C4H,ring	7.08(s)	119.8
		C2H,ring	7.85(s)	137.0
		COO(H)		176.8
**15**	Inosine	CH-ring	8.35(s)	143.2
		CH-ring	8.24(s)	155.6
		C1H-ribose	6.10(d)	91.2
		C2H-ribose	4.78	76.65
		C3H-ribose	4.44	b
		C4H-ribose	4.28	b
		C5H-ribose	3.87	b
**16**	Inosine-5'-monophosphate(IMP)	CH-ring	8.54(s)	142.5
		CH-ring	8.24(s)	149.1
		C1H-ribose	6.14(d)	89.9
		C2H-ribose	4.77	77.1
		C3H-ribose	4.51	73.2
		C4H-ribose	4.37	87.5
		C5H-ribose	4.03	66.2
**17**	Isoleucine (Ile)	α-CH	3.67(m)	62.3
		β-CH	1.98(m)	38.6
		γ-CH	1.27(m)	27.8
		γ'-CH	1.47(m)	27.8
		δ-CH_3	0.94(t)	13.9
		3- CH_3	1.01(d)	17.3
		COO(H)		176.4
**18**	Lactate	α-CH	4.11(q)	71.2
		β-CH_3	1.33(d)	22.7
		COO^-		185.2
**19**	Leucine(Leu)	α-CH	3.73(t)	56.0
		β-CH_2	1.72(m)	42.6
		γ-CH	1.69(m)	26.8
		δ-CH_3	0.97(d)	24.7
		δ'-CH_3	0.96(d)	23.7
		COO(H)		177.3
**20**	*Myo*-inositol	C1,3H	3.54(dd)	73.31
		C2H	4.06(t)	73.1
		C5H	3.26(t)	75.23
		C4,6H	3.62(t)	72.01
**21**	*N*-acetyl Aspartate	α-CH	4.39(dd)	54.01
		β-CH	2.68(dd)	40.54
		β'-CH	2.49(dd)	
		CH_3	2.02(s)	22.85
**22**	NAD	N5ring	8.20(m)	131.2
		N4ring	8.83(d)	148.7
		N2ring	9.34(s)	142.9

		N6ring	9.14(d)	145.2
		A2Hring	8.0(s)	126.0
		A8H ring	8.43(s)	143.3
		A4ring		151.2
		A1'H	6.09(d)	102.6
		N1'H	5.99	b
**23**	Oxidative Glutathione (GSSG)	S-CH2	3.314(dd)/2.98(dd)	41.3
		CH	4.76	55.4
		CH_2	3.76(m)	46
		CH_2	2.16(m)	29.2
		CH_2	2.51(m)	34.1
		C=O		177.8
		COOH		176.5
**24**	Phenylalanine (Phe)	α-CH	3.99(dd)	58.8
		β-CH	3.13(dd)	38.9
		β'-CH	3.27(dd)	38.9
		C1,ring		137.9
		C2,6,ring	7.33(m)	132.0
		C3,5,ring	7.42(m)	131.5
		C4,ring	7.38(m)	130.5
		COO(H)		176.7
**25**	Phosphocholine (PC)	α-CH_2	3.60(m)	69.5
		β-CH_2	4.17(m)	b
		N-CH_3	3.22(s)	56.7
**26**	Reduced Glutathione (GSH)	Glu α	3.78(t)	56.8
		Glu β	2.16(m)	29.2
		Glu γ	2.56(m)	34.1
		Cys α	4.57(dd)	58.6
		Cys β	2.94(m)	28.3
		Gly α	b	b
		C=O		174.5, 177.2
**27**	Succinate	CH_2	2.40(s)	35.9
		COO^-		183.6
**28**	Taurine	N-CH_2	3.27(t)	50.3
		S-CH_2	3.42(t)	38.2
**29**	Tyrosine (Tyr)	C3,5H,ring	6.90(d)	118.6
		C2,6H,ring	7.19(d)	133.3
		C1,ring		129.5
		α-CH	3.94(dd)	58.9
		β-CH_2	3.06	39.0
**30**	Uridine 5'-diphosphate (UDP)	CH-ring	7.99(d)	b
		CH-ring	5.97(d)	b
		C1H-ribose	5.96(broad,s)	b
		C3H-ribose	4.43(t)	b
		C2H-ribose	4.39(t)	b
		C4H-ribose	4.27(m)	b
		C5H-ribose	4.23(c)	b
**31**	UDP-glucose (UDPG)	G1-H	5.61	98.65
		C6,ring	7.96(d)	144.4
		C5,ring	5.98(d)	105.4

		C1’H,ribose	5.99(d)	91.3
		C2’3’H,ribose	4.38(m)	72.2/76.5
		C4’,ribose	4.29(m)	85.9
		C5’H,ribose	4.26/4.21(m)	67.8
		G2-H	3.90	73.1
		G6-H	3.86/3.78	63.36
		G3-H	3.77	74.7
		G4-H	3.54	74
		G5-H	3.47	72
**32**	UDP-*N*-acetyl glucosamine	C6,ring	7.96(d)	144.1
		C1’H,ribose	5.98(d)	91.1
		C5,ring	5.97(d)	105.4
		C2’3’H,ribose	4.37(m)	72.2
		C5’H,ribose	4.23/4.17(m)	66.2
		C4’H,ribose	4.29(m)	86.0
		C2,ring		156.6
		G1-H	5.52(dd)	97.2
		G2-H	3.99	56.5
		G3-H	3.82	73.7
		G4-H	3.55	72.2
		G5-H	3.93/3.867	
		NA-H	2.08	24.9
		NA-C=O		177.4
**33**	UDP-*N*-Acetyl Galactosamine	G1-H	5.55(dd)	97.4
		G2-H	4.05(m)	71.2
		G3-H	3.97(dd)	70.3
		G4-H	3.76(m)	57.4
		G5-H	3.79	63.6
		G6-H	3.78	63.6
		C2’3’H,ribose	4.37(m)	76.6
		C4’,ribose	4.29(m)	85.9
		C5’H,ribose	4.25/4.19(m)	67.8
		NA-H	2.089(s)	24.9
		C1’H,ribose	5.99(d)	91.1
		C5,ring	5.97(d)	105.4
		C6,ring	7.96(d)	144.1
**34**	Uracil	C5H	5.80(d)	b
		C6H	7.54(d)	b
**35**	Uridine	C6,ring	7.87(d)	144.1
		C1’H,ribose	5.92(d)	92.0
		C5,ring	5.90(d)	105.1
		C3’H,ribose	4.34(t)	72.2
		C5’H,ribose	4.12(q)	66.2
		C4’H,ribose	4.23(t)	86.0
		C2,ring		156.6
**36**	Valine (Val)	α-CH	3.61(d)	63.1
		β-CH	2.28(m)	31.9
		γ-CH_3	0.99(d)	19.5
		γ’-CH_3	1.04(d)	20.7
		COO(H)		176.8

Supplementary Table 2: Metabolites showed statistically significant differences in both cell line pairs between erlotinib-resistant and erlotinib-sensitive cells. "decrease" or "increase" meant statistically significant such differences were detected in the erlotinib-resistant cells as compared to sensitive ones. Keys refer to the peaks labelled on **Figure 1B and C**. The chemical shifts for the identified metabolites and the OPLS-DA coefficient for both cell line pairs are shown.

Key	Metabolite	Variation tendency	Chemical shift	Coefficient PC9 *vs* ER	Coefficient 1975 *vs* 3255
2	Adenosine	Decrease	6.087	0.81	0.72
4	Aspartate	Decrease	2.805	0.99	0.96
5	Choline	Increase	3.211	0.71	0.95
15	Inosine	Decrease	6.111	0.86	0.93
17	Isoleucine	Increase	1.019	0.99	0.87
19	Leucine	Increase	0.961	0.99	0.80
21	*N*-acetyl aspartate	Decrease	2.023	0.99	0.99
23	GSSG	Decrease	3.327	0.90	0.79
25	Phosphocholine	Increase	3.227	0.98	0.99
26	GSH	Decrease	4.571	0.96	0.69
30	UDP	Increase	7.995	0.96	0.93
31	UDPG	Increase	5.607	0.72	0.87
35	Uridine	Increase	5.909	0.87	0.68
36	Valine	Increase	1.053	0.97	0.87

Supplementary Figure 1

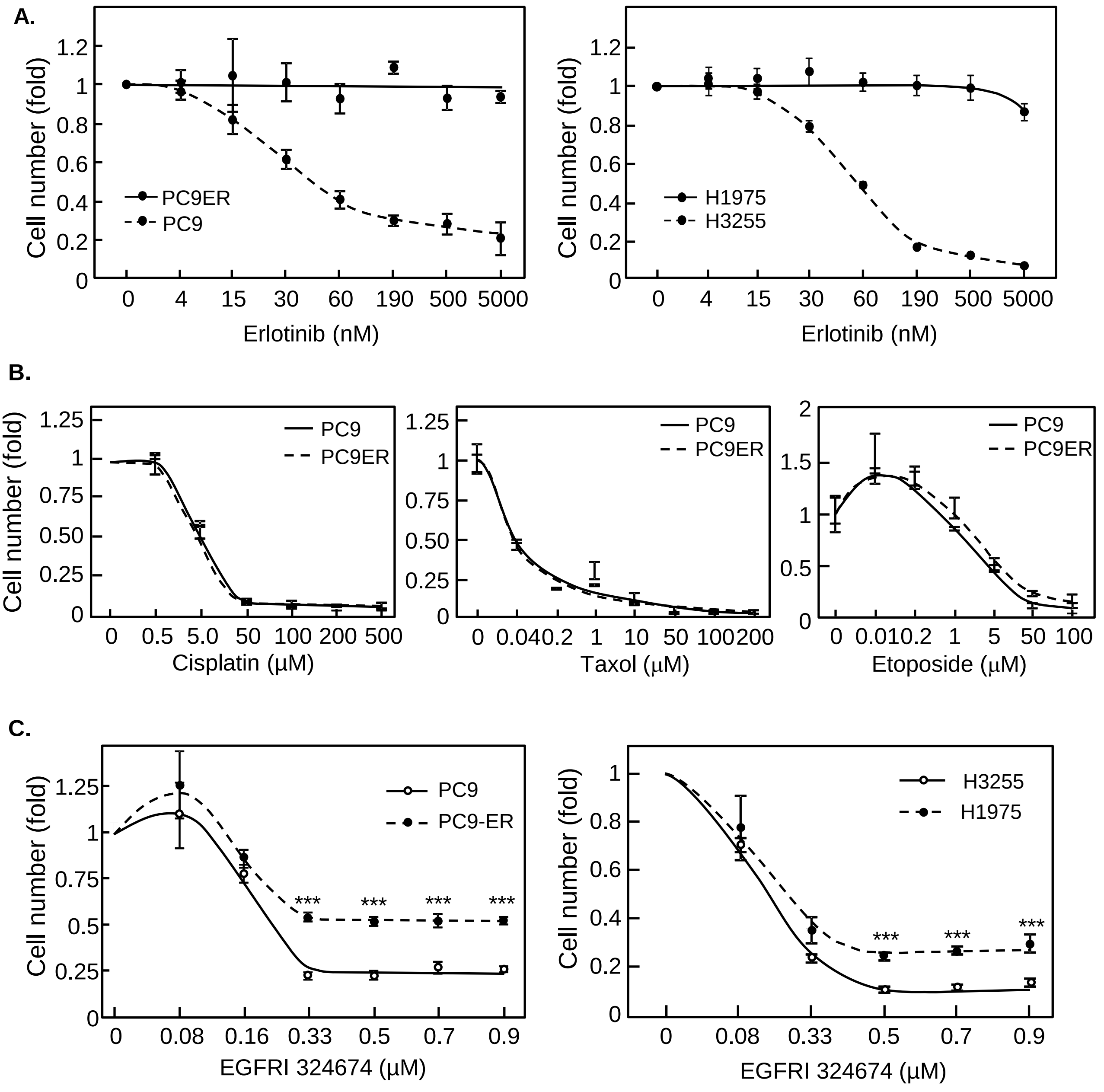

Supplementary Figure 2

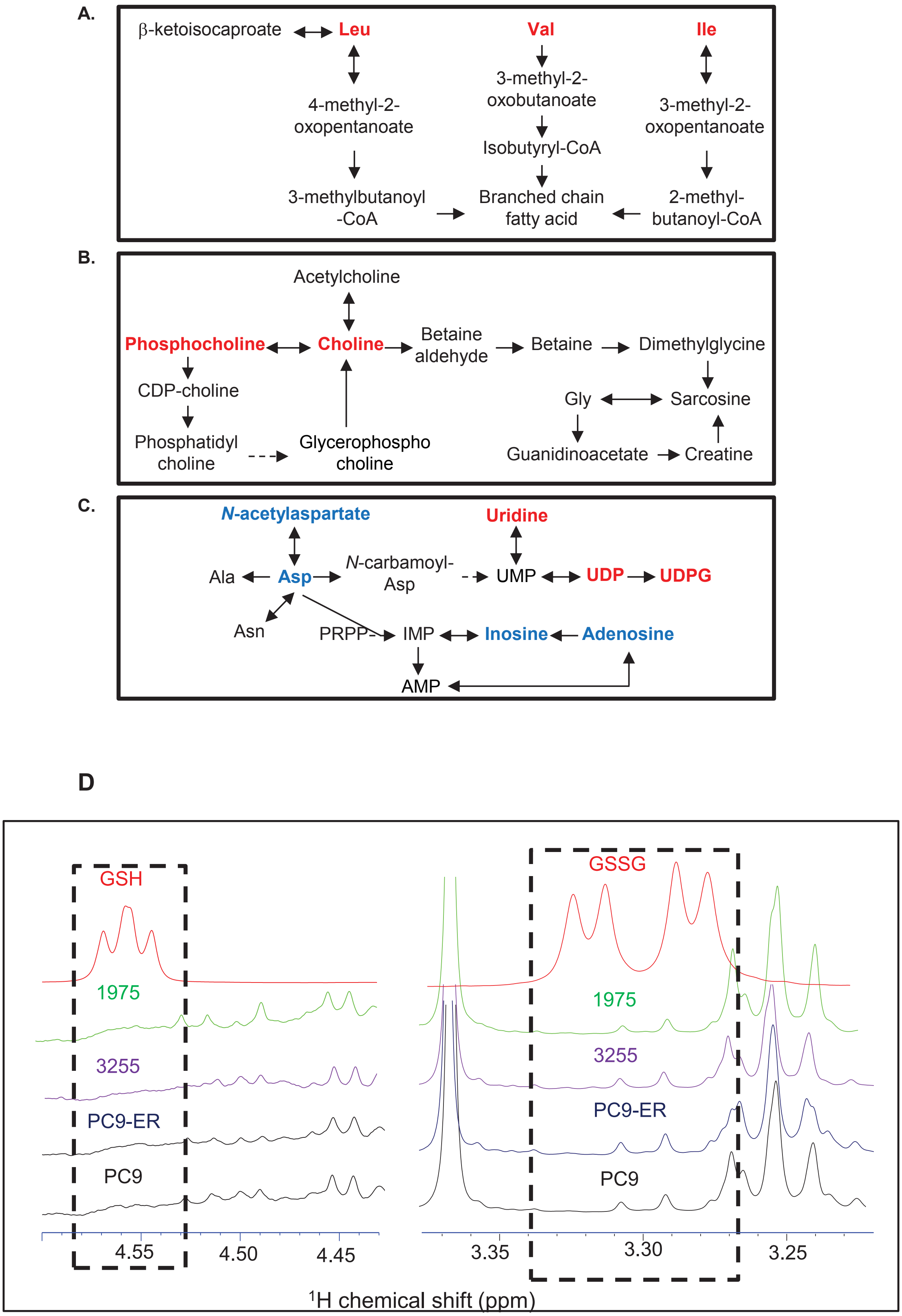

Supplementary Figure 3

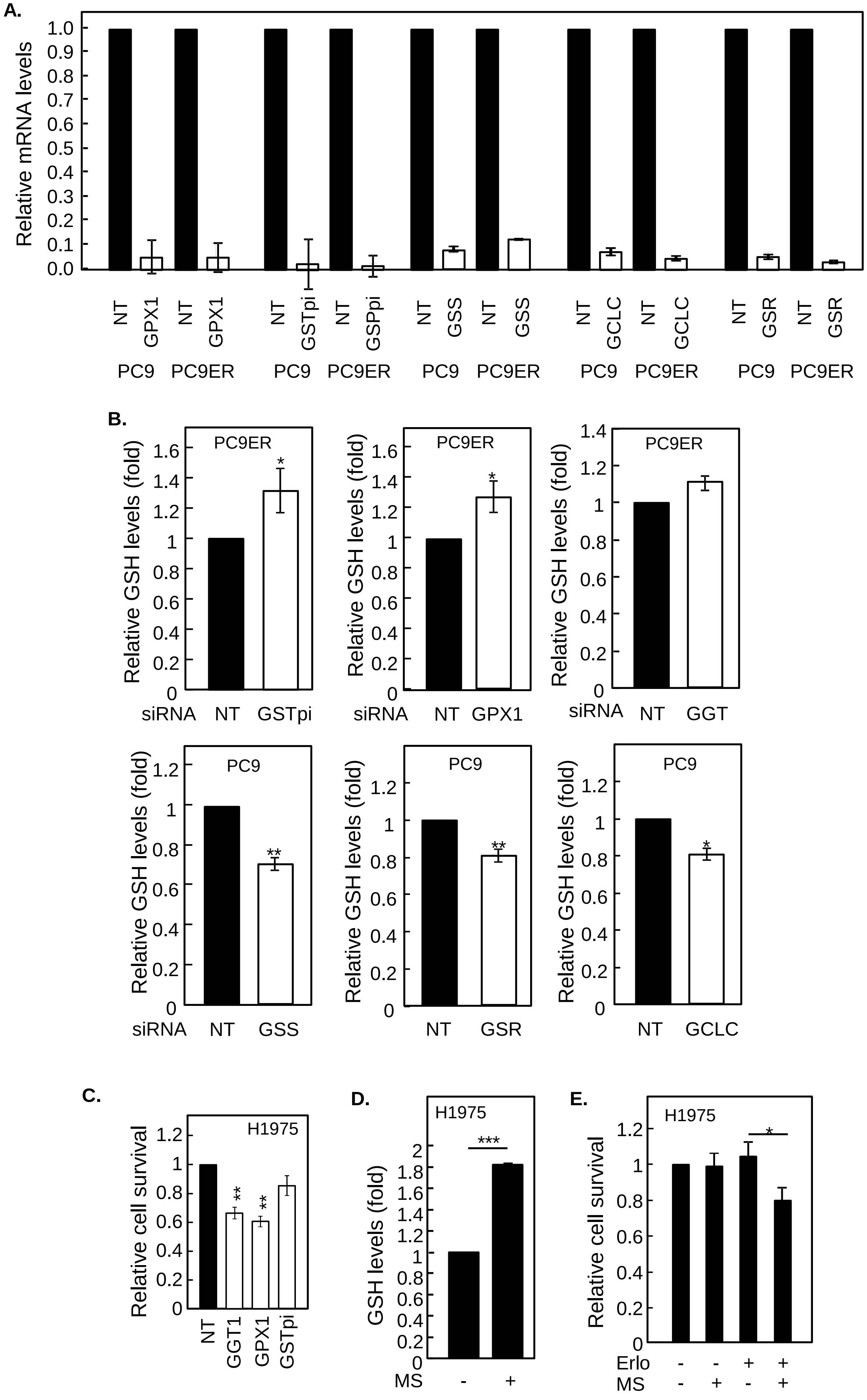

Supplementary Figure 4

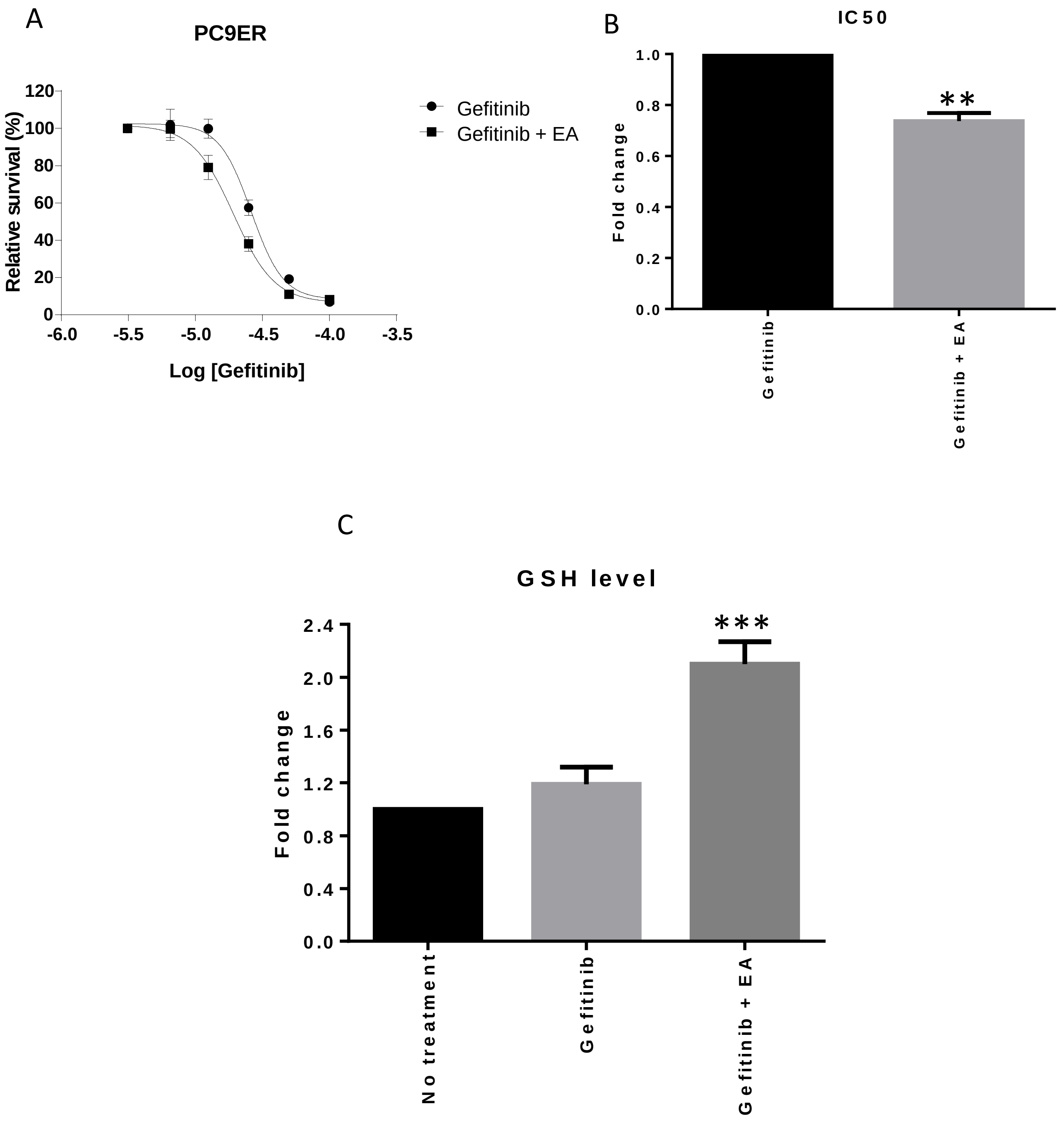

Supplementary Figure 5

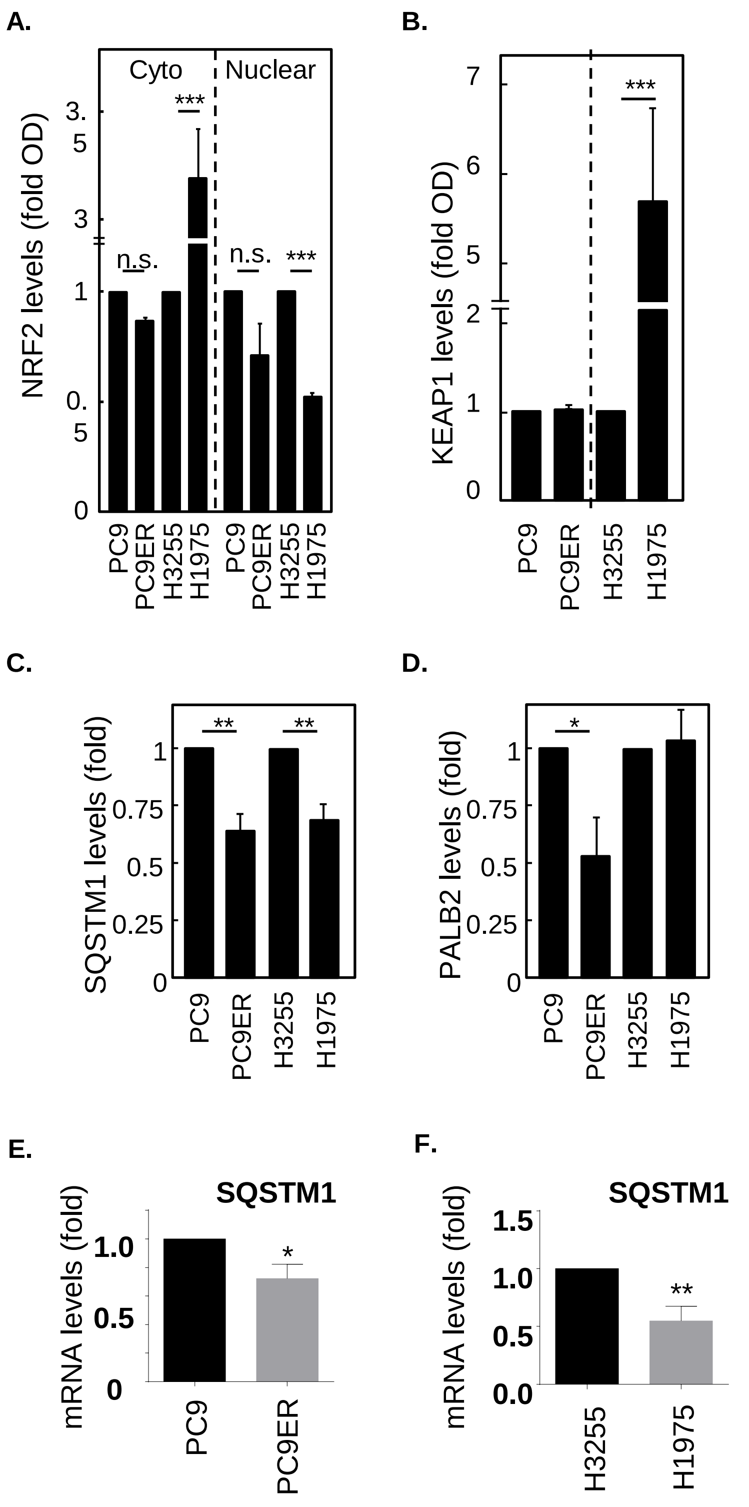

Supplementary Figure 6

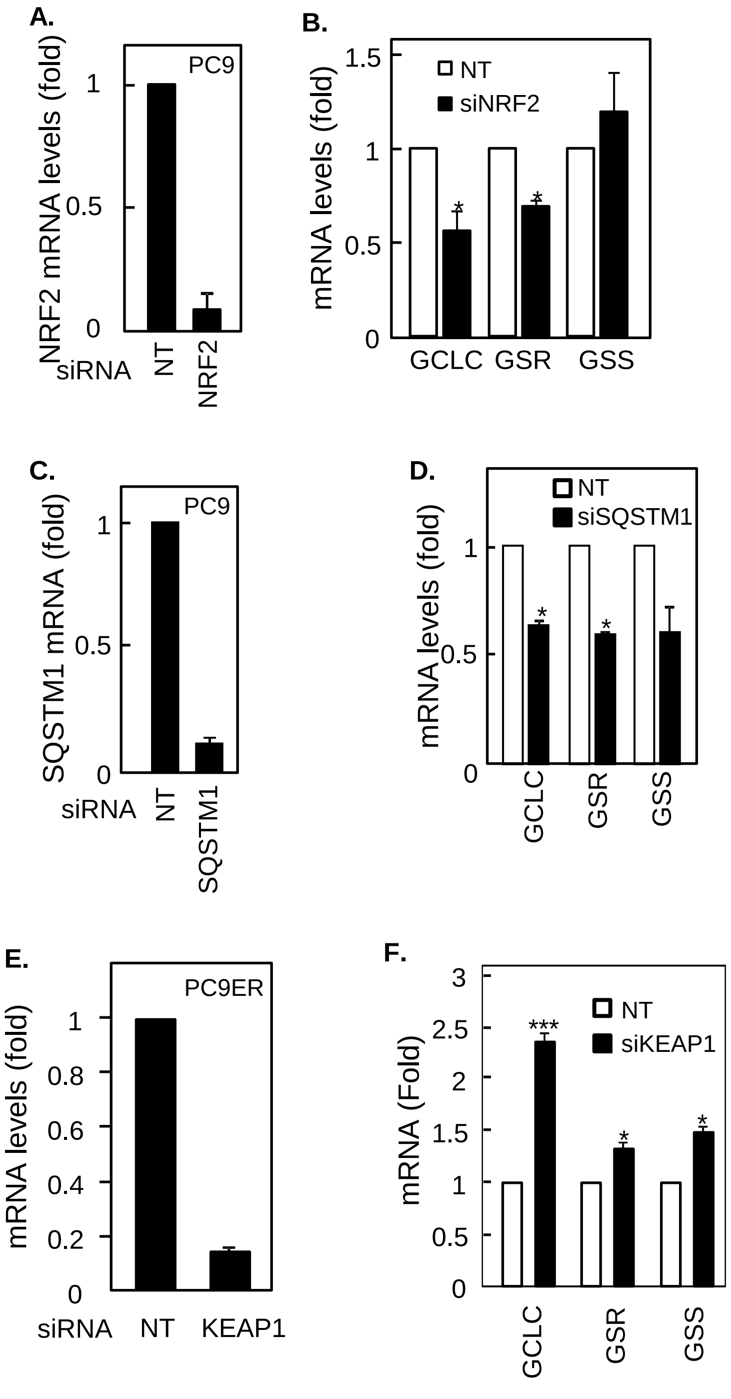

Supplementary Figure 7

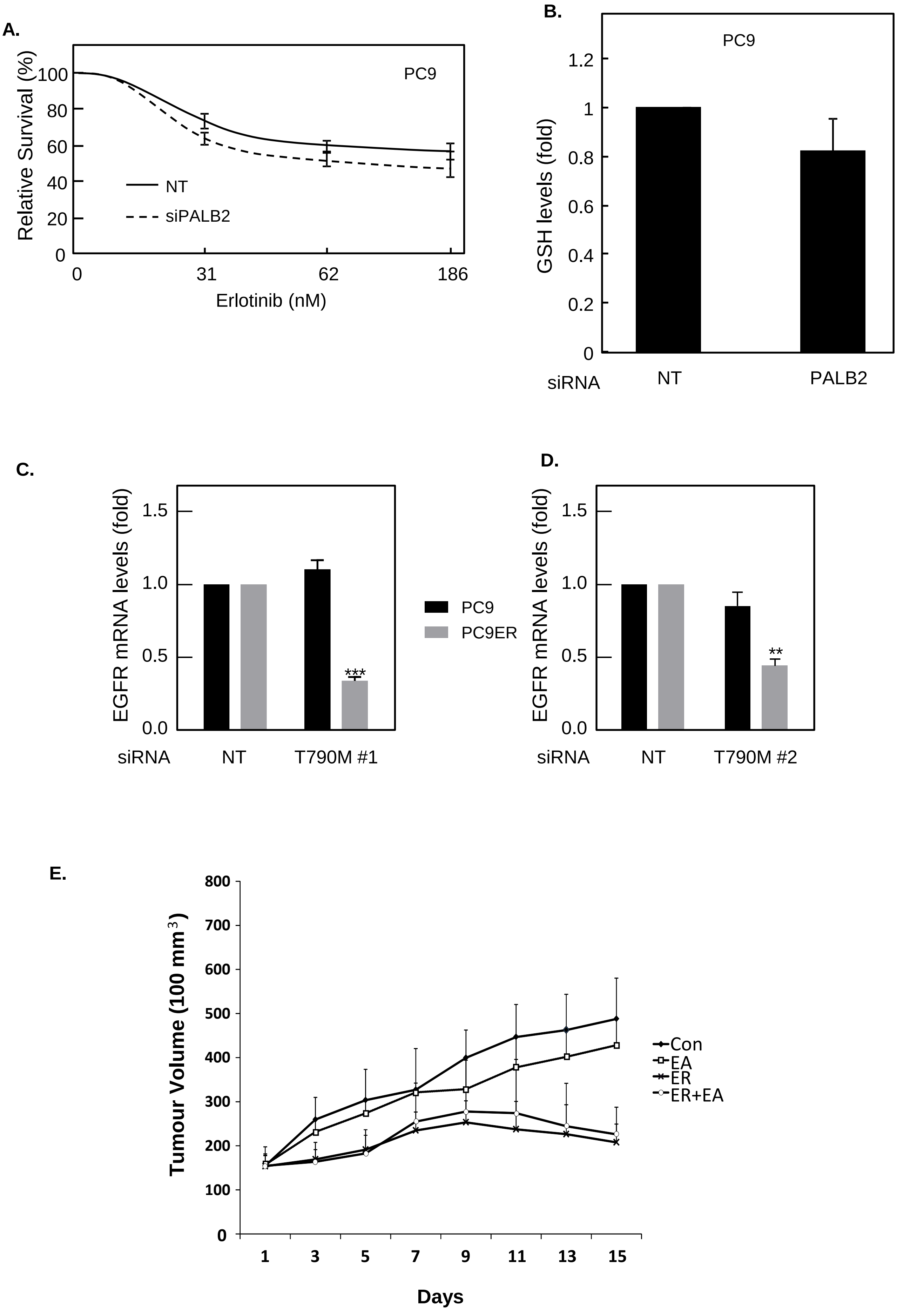

Supplement: Supplementary Information [file celldisc201631-s1.pdf]
